# Transformation of the Transcriptomic Profile of Mouse Periocular Mesenchyme During Formation of the Embryonic Cornea

**DOI:** 10.1167/iovs.18-26018

**Published:** 2019-02

**Authors:** Justin Ma, Peter Lwigale

**Affiliations:** BioSciences Department, Rice University, Houston, Texas, United States

**Keywords:** periocular neural crest, corneal development, corneal stroma, corneal endothelial cells, corneal epithelium

## Abstract

**Purpose:**

Defects in neural crest development are a major contributing factor in corneal dysgenesis, but little is known about the genetic landscape during corneal development. The purpose of this study was to provide a detailed transcriptome profile and evaluate changes in gene expression during mouse corneal development.

**Methods:**

RNA sequencing was used to uncover the transcriptomic profile of periocular mesenchyme (pNC) isolated at embryonic day (E) 10.5 and corneas isolated at E14.5 and E16.5. The spatiotemporal expression of several differentially expressed genes was validated by in situ hybridization.

**Results:**

Analysis of the whole-transcriptome profile between pNC and embryonic corneas identified 3815 unique differentially expressed genes. Pathway analysis revealed an enrichment of differentially expressed genes involved in signal transduction (retinoic acid, transforming growth factor-β, and Wnt pathways) and transcriptional regulation.

**Conclusions:**

Our analyses, for the first time, identify a large number of differentially expressed genes during progressive stages of mouse corneal development. Our data provide a comprehensive transcriptomic profile of the developing cornea. Combined, these data serve as a valuable resource for the identification of novel regulatory networks crucial for the advancement of studies in congenital defects, stem cell therapy, bioengineering, and adult corneal diseases.

Corneal development is a complex morphogenetic process that involves coordinated development of three distinct cellular layers, namely the epithelium, stroma, and endothelium, into a transparent tissue essential for vision. The formation of these distinct layers is interdependent and also governed by inductive signals from the surrounding ocular tissues that ensure proper cell migration, proliferation, and differentiation.^1^,^2^ The epithelium is derived from the ocular surface ectoderm, whereas the stromal keratocytes and endothelium are generated from the periocular mesenchyme that largely consists of a multipotent embryonic cell population, the neural crest.^3^–^5^ Four major events occur during mouse corneal development: (1) migration of periocular neural crest cells (pNC) into the presumptive corneal region, (2) differentiation of pNC into keratocytes and endothelium, (3) synthesis of stromal extracellular matrix (ECM) and formation of tight junctions and active pump function in the endothelium, and (4) maturation of the surface ectoderm into stratified corneal epithelium.^3^,^5^–^7^ Misregulation of the molecular cues that promote these events results in various forms of anterior segment dysgenesis.^8^–^10^

Major signaling pathways including retinoic acid (RA), transforming growth factor beta (TGFβ), and Wnt play critical roles during corneal development. RA is secreted by the optic cup and epithelium into the periocular mesenchyme, where it induces Foxc1 and Pitx2.^11^ This leads to activation of downstream effectors, such as Tfap2B and vascular endothelial growth factor, that are required for regulating cell fate and establishing angiogenic privilege.^12^,^13^ Mutations in the RA pathway leads to congenital anterior dysgenesis linked to Axenfeld-Rieger syndrome or Peters anomaly, characterized by corneal opacity and glaucoma.^14^,^15^ TGFβ is expressed by the lens epithelium,^16^ and it is required for pNC migration and differentiation into corneal endothelium.^16^–^18^ Although it is hypothesized that the maturation of corneal layers is interdependent, the effect of RA and TGFβ on epithelial maturation is not well studied. The Wnt and Notch signaling pathways are localized in the corneal epithelium where they regulate cell proliferation and stratification.^19^,^20^ Cross-talk between these signaling pathways regulates the expression of transcription factors, which play critical roles in imparting cellular identity and function,^21^ but the mechanisms involved are not well understood.

In this study, we used high-throughput RNA sequencing (RNA-Seq) to establish a transcriptome profile and analyze the changes in gene expression during mouse corneal development. We analyzed the downstream targets of RA, TGFβ, and Wnt signaling pathways and examined their combined effect on genes involved in modulating key processes, including ECM homeostasis, cell junctions, cell cycle, and neural vascular patterning. Our transcriptome data provide the first progressive expression signature that profiles the genetic landscape of the developing cornea. These findings increase our understanding of the fundamental molecular mechanisms that direct corneal development. In addition, this study identifies several novel genes that may play critical roles during corneal development, which may serve as potential targets for stem cell studies, bioengineering, and advancement of new corneal therapies.

## Materials and Methods

### Animals

Only wild-type C57/B6 mouse embryos were used for this study. All animal procedures were performed in accordance with the ARVO Statement for the Use of Animals in Ophthalmic and Vision Research and were approved by the Institutional Animal Care and Use Committee at Rice University. Timed pregnant mice were obtained from Jackson Laboratory, and embryos were collected at embryonic day (E) 10.5, E14.5, and E16.5 for tissue isolation and histology.

### Dissection of Periocular Mesenchyme and Embryonic Corneas

To obtain pNC, anterior eyes were dissected from E10.5 embryos, incubated in dispase (1.5 mg/ml; Worthington Biochemical, Lakewood, NJ, USA) at 37°C for 5 minutes, and then rinsed in Ringer's solution.The ectoderm/lens vesicles and optic cups were removed and discarded, and pNCs from 26 eyes were pooled into each sample. E14.5 corneas were dissected from surrounding ocular mesenchyme and pooled into 18 corneas per sample. Similarly, E16.5 corneas were dissected at the limbal region and pooled into 12 corneas per sample. Biological triplicates of tissues from each time point were immediately immersed in Trizol reagent (Life Technologies Corp., Grand Island, NY, USA) and flash frozen in liquid nitrogen.

### RNA Sequencing

RNA isolated from a total of nine samples was used for library preparation and sequenced on an Illumina HiSeq 4000 instrument at BGI Genomic Services, United States. Samples were qualified and quantified using an Agilent 2100 bioanalyzer and Step One Plus real-time PCR system. Each sample was assessed for quality by filtering out reads with adaptors, reads that contained a high percentage of unknown bases (>10%), or bases with low sequencing quality (Q < 5).^22^ The following reads were mapped to reference genes by Bowtie 2^23^ and to the Genome Reference Consortium Mouse Build 38 with Hierarchical Indexing for Spliced Alignment of Transcripts^24^ (Supplementary Table S1). The average mapping with the reference gene was 77.71%, and the genome mapping ratio was 91.47%. Reads were quantified using RNA-Seq by Expectation Maximization^25^ and normalized to fragments per kilobase of transcript per million (FPKM) to calculate gene expression levels. Aligned genes with no reads at a particular developmental stage were assigned a FPKM value of 0.01 for differential analysis. Screening of differentially expressed genes (DEGs) was performed through the NOISeq method^26^ by using the criteria of fold change of ≥1 and divergent probability of ≥0.8 (Supplementary Fig. S1). Deeper analysis into specific pathways followed stricter criteria. Based on log base 2 values, a threshold was set at 2.32 (FPKM = 5). Genes with all values below this threshold were considered not expressed. To reduce the uncertainty of low values, negative values were normalized to a base of 0 (FPKM = 1). Heatmaps were generated using log base 2 values with relative row scaling.

### Data Access

All sequencing data have been deposited in the NCBI's Gene Expression Omnibus database (https://www.ncbi.nlm.nih.gov/geo/browse/, in the public domain) under the accession number GSE121044.

### In Situ Hybridization

Section in situ hybridization was performed as previously described.^27^ In brief, mouse heads were isolated and fixed in Carnoy's fixative at 4°C overnight. Tissues were embedded in paraffin and sectioned at 8 to 10 μm. Digoxigenin-labeled riboprobes were generated by in vitro transcription with Superscript III. Brightfield images were captured using a Zeiss Axiocam mounted on AxioImager2 microscope (Zeiss, Oberkochen, Baden-Württemberg, Germany).

## Results

### Characterization of the Transcriptomes of pNC and Embryonic Corneas

**Figure 1:**
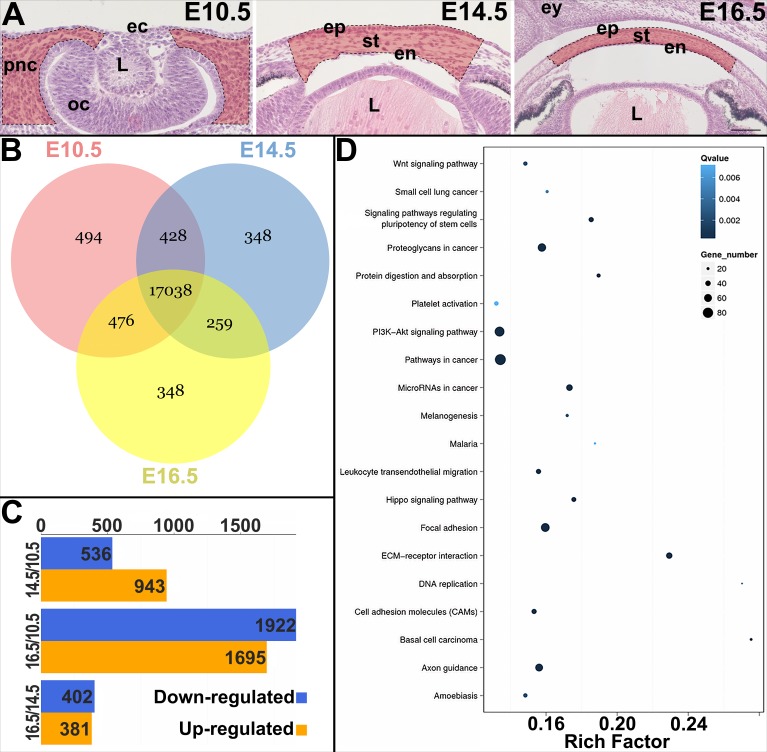
RNA sequencing analysis of the pNC and embryonic corneal cells. (A) Hematoxylin and eosin staining of mouse embryonic eyes, with highlighted regions within the dotted lines showing the tissue dissected for RNA preparation at E10.5, E14.5, and E16.5. (B) Venn diagram depicts total number of genes categorized between the three stages. (C) Bar plot showing number of differentially regulated genes detected between E10.5 and E14.5, E10.5 and E16.5, and E14.5 and E16.5. (D) Pathway enrichment analysis of DEGs at E10.5 vs. E14.5. Circle size correlates with number of genes and the Rich factor is a representation of the degree of enrichment based on ratio of DEG/non-DEG within the pathway. Scale bars: 50 μm (E10.5 and E14.5) or 100 μm (E16.5). ec, ectoderm; L, lens; oc, optic cup; ep, epithelium; st, stroma; en, endothelium; ey, eyelid.

To investigate the transcriptomic profile during corneal development, we performed high-throughput RNA-Seq on pNC isolated at E10.5 and embryonic corneas isolated at E14.5 and E16.5 (Fig. 1A). These time points were selected to capture pNC migration into the corneal region (E10.5), differentiation of corneal epithelium and pNC-derived mesenchyme (E14.5), and postformation of the three cellular layers of the cornea (E16.5).

RNA-Seq analysis generated an average of 23,029,819 raw reads. Alignment of reads identified transcripts for 19,391 unique genes, of which reads for 17,038 were detected at all 3 developmental stages (Fig. 1B). Categorizing the transcripts using the NOISeq method revealed 3815 unique DEGs. A total of 1479 genes were differentially expressed between E10.5 and E14.5, of which 536 were downregulated and 943 were upregulated (Fig. 1C). Analysis between E10.5 and E16.5 yielded 3617 DEGs, of which 1922 were downregulated and 1696 were upregulated. We also compared E14.5 and E16.5, which showed that 783 genes were differentially expressed, of which 402 were downregulated and 381 were upregulated. Overall, there was a high number of DEGs between E10.5 and E16.5, which substantially decreased between E10.5 and E14.5, and E14.5 and E16.5 (Fig. 1C). This is supported by hierarchical clustering that indicates higher similarity in transcriptome between E14.5 and E16.5 compared to E10.5 and E14.5 or E10.5 and E16.5 (Supplementary Fig. S1). Further analyses show that 506 genes were enriched only at E10.5, 71 at E14.5, and 355 at E16.5.

To associate the DEGs to functional roles, we analyzed their distribution by using pathway enrichment analysis based on the KEGG database (Fig. 1D). Several key pathways and processes were significantly enriched, including focal adhesions, ECM-receptor interactions, proteoglycans, and cell adhesion molecules. These pathways and cell processes are important in mediating pNC migration, cell proliferation, matrix assembly, and modulating barrier functions.

### Regulation of Neural Crest Cell (NCC) Markers During Corneal Development

**Figure 2:**
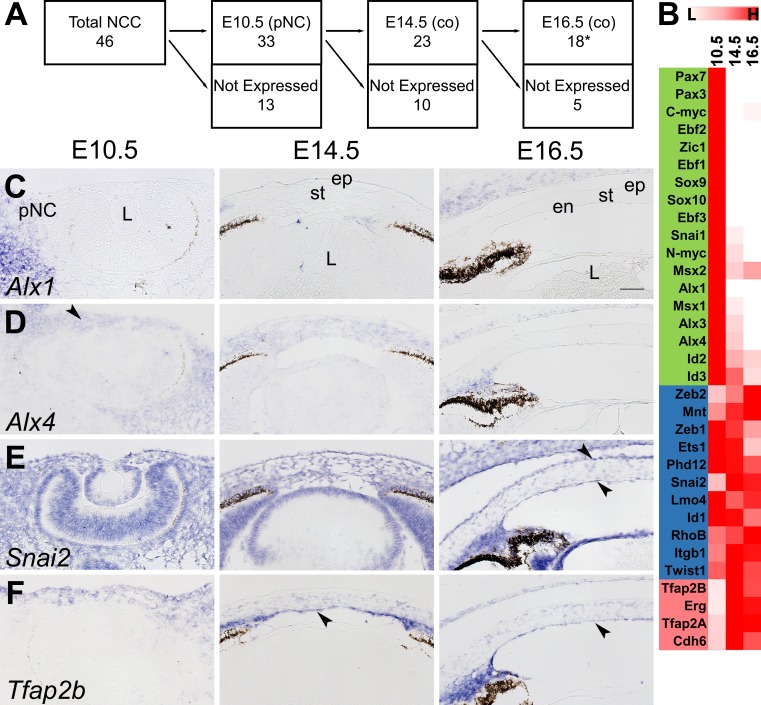
Expression of NCC genes during corneal development. (A) Schematic describes the number of expressed genes at each developmental stage based on threshold value. (B) Heatmap shows relative expression of the transcripts in the pNC, E14.5, and E16.5 corneas. Relative color ranges from white to red based on low (L) or high (H) expression. In addition to the criteria described in the methods, values below threshold were normalized to a log base 2 value of 0. Downregulated genes are highlighted in green, not significantly DEGs in blue, upregulated genes in red, and genes below threshold are not shown (see Supplementary Table S2). (C–F) Validation of the expression patterns of Alx1, Alx4, Snai2, and Tfap2b. Black arrows represent regions of enriched expression. Scale bar: 50 μm. co, Cornea; *C-myc expression at E16.5 is excluded.

To determine whether genes that are important for establishing NCC identity continue to play a role during corneal development, we analyzed the expression of 46 candidate genes involved in NCC specification, delamination, and early migration.^28^,^29^ Based on our threshold value of FPKM of 5, we found that out of the 46 genes, 33 (72%) re-expressed in the pNC, 23 (50%) in the E14.5 corneas, and 18 (39%) in the E16.5 corneas (Fig. 2A). Classification of the 46 NCC genes based on differential regulation (Fig. 2B), revealed that 18 (39%) of genes, including *Alx1*, *Alx4*, *Pax3*, *Pax7*, *Zic1*, *Zic2*, *Sox9*, and *Sox10*, are enriched in the pNC. Eleven (24%) genes, including *Zeb1*, *Zeb2*, *Snai2*, *Lmo4*, and *Twist1*, maintained nondifferential expression. Four (9%) genes (*Tfap2A*, *Tfap2B*, *Erg*, and *Cdh6*) are upregulated in the cornea, whereas the remaining 13 (28%) genes, including *Axud1*, *Foxd3*, *Gbx2*, and *Rxrg*, are not expressed (Supplementary Table S2). To validate our data, we analyzed the spatiotemporal expression of *Alx1*, *Alx4*, *Snai2*, and *Tfap2B* by in situ hybridization. *Alx1* is expressed in the pNC at E10.5, but it is not detected in the corneas at E14.5 and E16.5 (Fig. 2C). *Alx4* is expressed in the pNC at E10.5 and stroma at E14.5 but absent in the cornea at E16.5 (Fig. 2D). *Snai2* is broadly expressed at all time points and shows strong localization to the corneal epithelium and endothelium at E16.5 (Fig. 2E). *Tfap2b* is initially expressed in a few pNC cells and ocular ectoderm at E10.5, but it is strongly expressed in the corneal stroma and endothelium at E14.5 and E16.5 (Fig. 2F).

### Regulation of RA Signaling During Corneal Development

**Figure 3:**
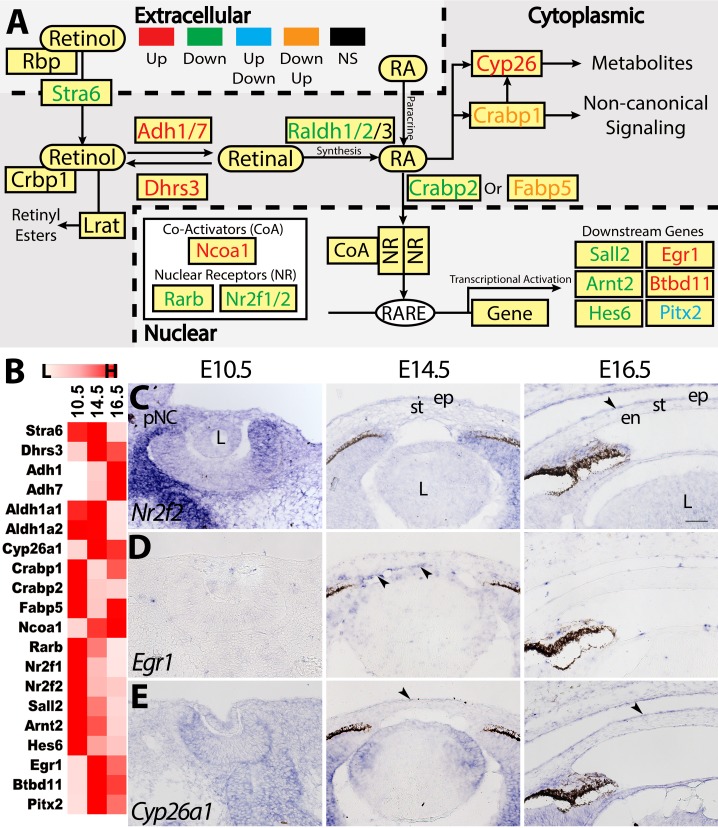
Differential regulation of the RA signaling pathway. (A) Schematic depicts whether components of the RA pathway are upregulated (red), downregulated (green), or not significantly differentially expressed (black). Genes that were upregulated and then downregulated, or vice versa, are represented by blue and orange, respectively. (B) Heatmap summarizes the relative expression of the DEGs. (C–E) Validation of the expression patterns of Nr2f2, Egr1, and Cyp26a1. Black arrows represent regions of enriched expression. Scale bar: 50 μm.

We investigated changes to the RA signaling components and found that genes important for metabolism and signaling are differentially regulated (Fig. 3A; Supplementary Table S3).^30^–^32^ Prometabolic genes, such as *Stra6*, *Raldh1*, and *Raldh2*,^33^ are not significantly changed between E10.5 and E14.5, but they are downregulated at E16.5. In contrast, *Adh1* and *Adh7* are upregulated at E16.5. *Dhrs3*, a metabolic inhibitor that converts retinal back into retinol,^33^ is upregulated at E14.5. *Raldh3* is constitutively expressed at high levels, but its expression is localized to the corneal epithelium.^34^ The RA-degrading enzyme *Cyp26a1*^35^ is upregulated at E14.5. *Crabp2*, which translocates RA from the cytoplasm into the nucleus,^30^ is downregulated, whereas *Crabp1* and *Fabp5* are downregulated at E14.5 but upregulated at E16.5. A majority of the nuclear receptors, including *Rara*, *Rarg*, *Rxra*, *Rxrb*, *Nr1h2*, and *Ppard*, are constitutively expressed, but *Rarb*, *Nr2f1*, and *Nr2f2* are downregulated (Fig. 3B; Supplementary Table S3). Corresponding with these changes, several RA-responsive transcription factors (*Sall2*, *Arnt2*, *Hes6*, and *Pitx2*)^36^–^39^ are downregulated at E16.5 (Fig. 3B). RA-induced genes (*Egr1* and *Btdbd11*)^40^,^41^ are also substantially decreased at E16.5 (Fig. 3B; Supplementary Table S3). To identify the corneal regions in which RA signaling is regulated, we examined the expression profiles of an RA inhibitor, *Cyp26a1*, a nuclear receptor, *Nr2f2*, and a downstream gene, *Egr1* (Figs. 3C–E). Our data show that *Cyp26a1* is broadly expressed at all time points, with strong localization in the corneal epithelium at E14.5 and E16.5 (Fig. 3C). *Nr2f2* is strongly expressed in the pNC at E10.5 and maintained at low levels in the stroma, but it is localized in the corneal epithelium at E14.5 and E16.5 (Fig. 3D). *Egr1* is not detectable in the pNC and cornea at E16.5, but it is transiently expressed in the presumptive corneal endothelium at E14.5 (Fig. 3D).

### Regulation of TGFβ Signaling During Corneal Development

**Figure 4:**
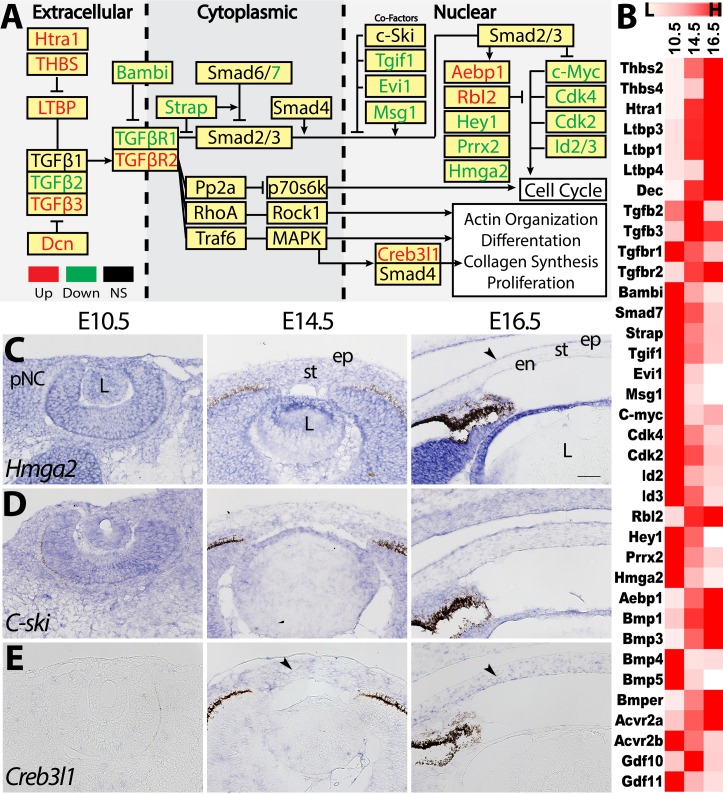
Differential regulation of the TGFβ signaling pathway. (A) Schematic depicts whether components of the TGFβ pathway are upregulated (red), downregulated (green), or not significantly differentially expressed (black). (B) Heatmap summarizes the relative expression of the DEGs. (C–E) Validation of the expression patterns of Hmga2, C-ski, and Creb3l1. Black arrows represent regions of enriched expression. Scale bar: 50 μm.

To examine the mechanisms by which TGFβ signaling regulates corneal development, we investigated the transcription profile of its ligands and downstream genes (Fig. 4A; Supplementary Table S4).^42^,^43^ Our data show that *TGFβ2* is strongly expressed at E10.5 and E14.5 but downregulated at E16.5, and *TGFβ3* is upregulated at E14.5 and E16.5. Interestingly, *TGFβR2* is upregulated at E14.5 and E16.5, but its associated receptor *TGFβR1*^42^ is downregulated. In addition, multiple inhibitors (*Bambi*, *Strap*, *Smad7*, *Tgif*, and *Evi1*) and an activator (*Msg1*) of TGFβ signaling through Smad2/3 regulation are downregulated. Overall, a large number of DEGs favors enrichment of the TGFβ pathway. Accordingly, genes repressed by the TGFβ pathway (*Cdk2*, *Cdk4*, *C-myc*, *Id2*, and *Id3*) are downregulated, and TGFβ-induced genes (*Rbl2*, *Aebp1*, and *Creb3l1*) are upregulated. The observed differential regulation aligns with TGFβ function in cell cycle regulation, differentiation, and ECM synthesis.^44^–^49^ We also observed that several TGFβ-induced epithelial-mesenchymal transition genes, including *Hey1* and *Prrx2*,^50^,^51^ were downregulated, possibly due to regulation through other pathways.

To analyze how TGFβ regulates corneal development, we examined the expression of downstream targets *Hmga2*,^52^ nuclear repressor *c-Ski*,^53^ and mediator of collagen synthesis *Creb3l1*^48^ (Figs. 4C–E). The observed expression patterns are consistent with our dataset and show that *Hmga2* is initially ubiquitously expressed at E10.5 and E14.5, but it localizes to the corneal epithelium at E16.5 (Fig. 4C). *c-Ski* is constitutively expressed in the pNC and cornea (Fig. 4D). *Creb3l1* is not expressed at E10.5, but it was strongly expressed in the corneal stroma and endothelium at E14.5 and E16.5 (Fig. 4E). In addition to the changes observed in the canonical TGFβ signaling, we discovered differential regulation of other members of the TGFβ superfamily. *Bmp4*, *Bmp5*, *Acvr2b*, and *Gdf11* are all downregulated, whereas *Bmp1*, *Bmp3*, *Avcr2a*, and *Gdf10* are upregulated (Fig. 4B).

### Regulation of the Wnt Signaling During Corneal Development

**Figure 5:**
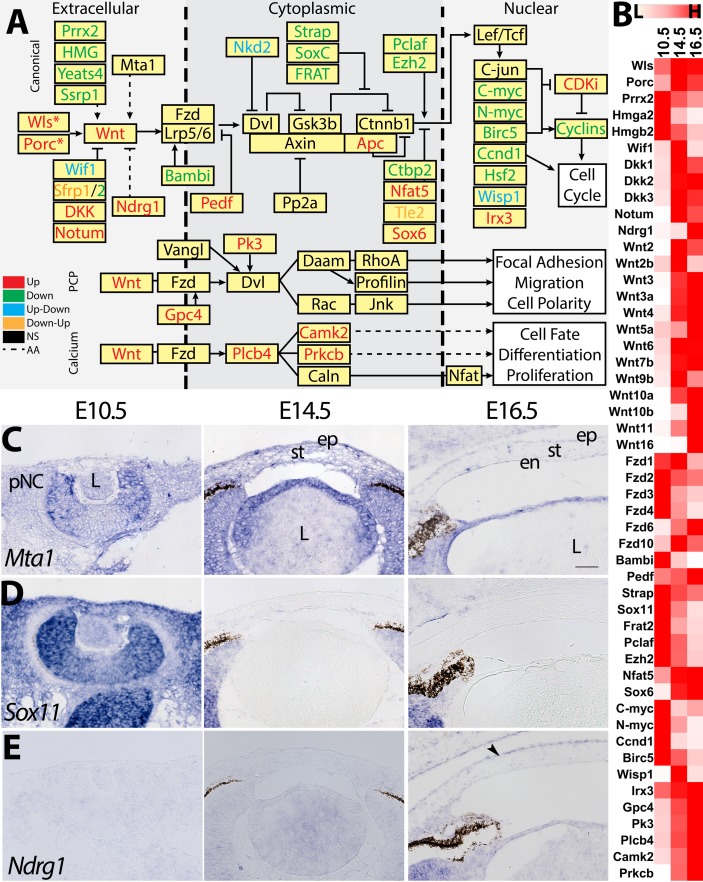
Differential regulation of the Wnt signaling pathway. (A) Schematic depicts whether components of the Wnt pathway are upregulated (red), downregulated (green), or not significantly differentially expressed (black). (B) Heatmap summarizes the relative expression of the DEGs. (C–E) Validation of the expression patterns of Mta1, Sox11, and Ndrg1. Black arrows represent regions of enriched expression. Scale bar: 50 μm. AA, ambiguously associated.

Next, we investigated the mechanisms by which the Wnt pathway is modulated during corneal development (Fig. 5A).^54^,^55^ Our data reveal that several Wnt genes (*Wnt2*, *Wnt2b*, *Wnt3*, *Wnt3a*, *Wnt4*, *Wnt5a*, *Wnt6*, *Wnt7b*, *Wnt9b*, *Wnt10a*, *Wnt10b*, *Wnt11*, and *Wnt16*) are upregulated (Fig. 5B). However, Frizzled receptors are either upregulated (*Fzd6* and *Fzd10*) or downregulated at E14.5 (*Fzd3* and *Fzd4*) and E16.5 (*Fzd1* and *Fzd2*) (Fig. 5B; Supplementary Table S5). This is consistent with previous reports^56^ and further identifies the novel expression of Wnt ligands and receptors. We found that many inducers of canonical activity, including *Prrx2*, HMG family, *Bambi*, *Strap*, *Sox11*, *Frat2*, *Pclaf*, and *Ezh2* are downregulated, whereas the repressors *Wif1*, *Dkk1*, *Dkk2*, *Dkk3*, *Notum*, *Ndrg1*, *Nfat5*, and *Sox6* are upregulated (Figs. 5A, 5B). Spatiotemporal analysis confirmed the expression of candidate Wnt modulators. The Wnt activator *Mta1*^57^ is localized in the periocular mesenchyme and all cellular layers of the cornea (Fig. 5C), whereas the Wnt activator *Sox11*^58^ is initially strongly expressed in the pNC at E10.5, but it is not detectable at E14.5 and E16.5 (Fig. 5D). The upregulated Wnt inhibitor *Ndrg1*^59^,^60^ is not detectable in the pNC at E10.5, but it is later localized to the corneal epithelium at E16.5 (Fig. 5E).

Next, we analyzed how Wnt downstream genes are modulated. Our data revealed that several downstream targets, particularly those related to proliferation (*C-myc*, *N-myc*, *Ccnd1*, and *Birc5*) were downregulated (Figs. 5A, 5B). However, we also identified upregulation of a few genes that are activated by the canonical pathway (*Wisp1* and *Irx3*). Our data also indicate that genes involved in the Wnt/planar cell polarity (PCP) and Wnt/Ca^2+^ pathways were upregulated (*Gpc4*, *Pk3*, *Plcb4*, *Camk2*, and *Prkcb*).

### Crosstalk Between Signaling Pathways Is Critical for Corneal Development

**Figure 6:**
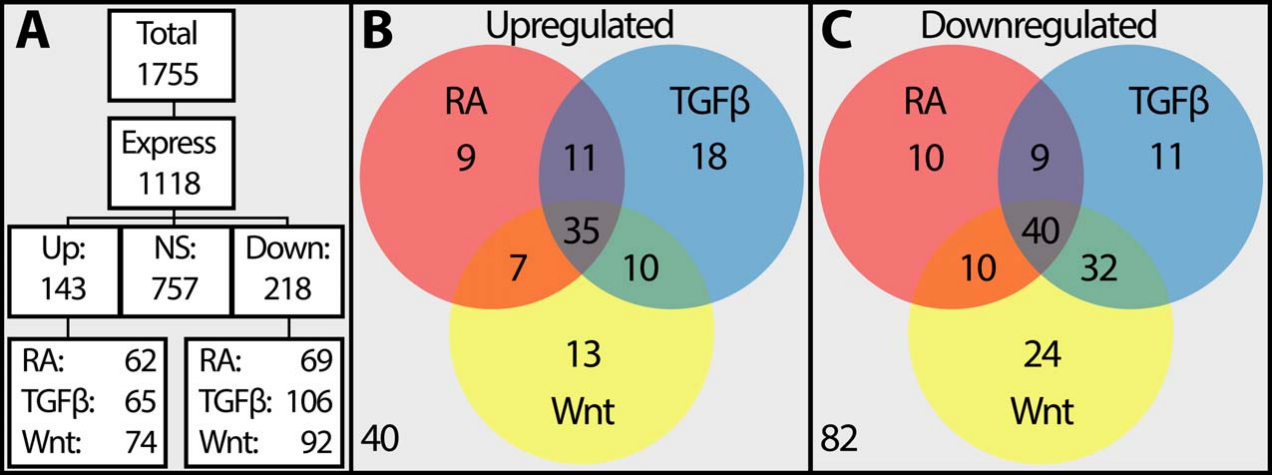
Categorization of differentially expressed transcription factors into the RA, TGFb, and Wnt signaling pathways. (A) Allocation of transcription factors from a total pool of 1755 (compiled from Riken's mouse database and self-annotated). (B, C) Venn diagrams showing overlap between differentially expressed transcription factors. Values outside the circles represent the genes that are not well characterized or not studied within the relevant pathways.

To examine how the cross talk between RA, TGFβ, and Wnt signaling pathways regulates corneal development, we analyzed the differential expression of their downstream transcription factors. Out of 1755 transcription factors, we found a total of 1118 genes expressed above the threshold. Of these genes, 143 were upregulated, 218 were downregulated, and 757 were not differentially expressed (Fig. 6A). Next, we annotated their association with the signaling pathways based on published data. From those upregulated genes, 62 are associated with RA signaling, 65 with TGFβ signaling, 74 with Wnt signaling, and the data are insufficient for 40. From those downregulated genes, 69 are associated with RA signaling, 92 with TGFβ signaling, 106 with Wnt signaling, and the data are insufficient for 82. The top 20 upregulated and downregulated transcription factors are summarized in Table 1 and are in a full list in Supplementary Table S6. Several transcription factors, such as Pax6 or Foxc2, are involved in more than one pathway, indicating potential cross talk during corneal development.

**Table 1:** Top Differentially Expressed Transcription Factors

**Symbol**	**14.5/10.5**	**16.5/10.5**	**16.5/14.5**	**RA**	**TGFβ**	**Wnt**
Upregulated (Log2)						
Fosl2	4.86	6.46	1.59	✓	✓	
Klf5	3.84	5.78	1.94	✓	✓	✓
Ahr	4.93	5.26	0.33	✓	✓	✓
Pax6	3.85	4.77	0.92	✓	✓	✓
Pou3f3	4.62	4.73	0.12			
Creb3l1	3.50	4.71	1.22		✓	
Rab25	2.77	4.71	1.94	✓	✓	✓
Bcl6	3.14	4.61	1.47	✓	✓	✓
Hlf	3.06	4.53	1.47		✓	
Ankrd3	3.15	4.08	0.93			✓
Ehf	1.11	4.07	2.96		✓	
Nupr1	2.17	4.03	1.86	✓	✓	✓
Otx1	3.93	3.61	−0.32	✓		
Trp73	2.76	3.92	1.16	✓	✓	
Erg	3.92	3.72	−0.20	✓	✓	✓
Nr3c1	3.35	3.90	0.54			
Ptrf	2.69	3.73	1.04		✓	
Tcfap2b	3.72	2.83	−0.89	✓	✓	✓
Cebpa	2.93	3.70	0.77	✓	✓	✓
Irx4	3.62	3.36	−0.26	✓		
Downregulated (Log2)						
Six2	−6.10	−6.06	0.04	✓	✓	✓
Msx1	−2.91	−5.88	−2.96	✓	✓	✓
Cart1	−5.81	−5.81	0.00	✓		
Foxl2os	−5.01	−5.42	−0.41			
Foxl2	−5.17	−5.22	−0.05		✓	✓
Foxd1	−5.14	−5.14	0.00	✓	✓	✓
Foxc2	−2.85	−4.93	−2.07	✓	✓	✓
Hmga2	−2.78	−4.93	−2.14		✓	✓
Ebf3	−4.24	−4.88	−0.64			
Six1	−4.86	−4.86	0.00		✓	✓
Gli	−1.60	−4.80	−3.20		✓	✓
Barx1	−4.51	−4.51	0.00			✓
Asb4	−3.41	−4.17	−0.77			
Foxd2	−3.52	−4.11	−0.59			✓
Zic2	−4.02	−4.02	0.00	✓	✓	✓
Alx4	−2.03	−4.01	−1.98	✓	✓	✓
Foxf2	−3.49	−3.95	−0.46	✓	✓	✓
Alx3	−2.03	−3.91	−1.88		✓	✓
Arid3b	−3.01	−3.91	−0.90			✓
Foxp2	−3.50	−3.89	−0.39	✓		✓

Checkmarks indicate potential association with signaling pathways based on published data.

The net regulatory effect of the above transcription factors determines corneal morphogenesis. This includes formation of the collagen ultrastructure, proliferation and differentiation of the cellular layers, and neurovascular patterning.^5^,^6^,^61^ Therefore, we analyzed the expression of critical components of corneal development, including genes for the ECM, matrix remodeling proteins, ECM receptors, cell junction proteins, epithelial development, cell cycle, and neurovascular patterning. The top DEGs are reported in Tables 2 to 5 and full lists are in Supplementary Tables S7 to S14. Our data indicate that the majority of the ECM and matrix remodeling proteins, including collagens, laminins, and thrombospondins, are upregulated and expressed at high levels (Table 2, Supplementary Tables S7–S9), indicating that a large number of components contribute to establishing the ultrastructure. We also observed downregulated genes, such as *Vtn*, *Emilin2*, and *Nid2*, that may play critical roles during early corneal development. In addition, several extracellular matrix receptors are upregulated (*Itga11*, *Itga3*, *ItgaV*, *Itgb4*, *Dag1*, *Ddr1*, and *Cd44*) or downregulated (*Itga4*, *Itga8*, and *Itga9*) (Table 2). Although not differentially expressed, transcripts for *Itga5*, *Itga6*, *Itgb1*, and *Itgb5* are detected at high levels (Supplementary Table S10), and they may form heterodimers with differentially expressed integrins.^62^ Expression of cell junction genes, such as *Gja1*, *Tjp1*, and *Ocln*, are similarly enriched (Table 2, Supplementary Table S11). This is accompanied by the expression of genes involved in differentiation of the corneal epithelium, including *Pax6*, *Klf4*, and *Klf5*,^63^–^66^ as well as epithelial structural genes, such as *Krt5*, *Krt12*, *Krt14*, and *Krt15*^67^ (Table 3, Supplementary Table S12).

**Table 2:** Top Differentially Expressed ECM and Junction-Associated Genes

**Upregulated (Log2)**				**Downregulated (Log2)**			
**Symbol**	**14.5/10.5**	**16.5/10.5**	**16.5/14.5**	**Symbol**	**14.5/10.5**	**16.5/10.5**	**16.5/14.5**
ECM-core matrisome (no collagen)							
Kera	8.43	10.25	1.81	Smoc1	−3.64	−3.64	0.00
Dcn	7.41	8.54	1.12	Tgfbi	−3.23	−0.99	2.25
Matn4	7.68	8.27	0.59	Vtn	−3.11	−3.11	0.00
Thbs4	2.38	6.06	3.68	Igfbp3	−0.25	−2.79	−2.53
Podn	5.18	6.04	0.86	Mmrn2	−0.88	−2.73	−1.85
Papln	1.72	5.98	4.26	Hapln1	−0.77	−2.66	−1.90
Thbs2	4.09	5.95	1.86	Mmrn1	−2.53	−2.61	−0.08
Smoc2	4.78	5.29	0.51	Emilin2	−0.64	−2.52	−1.89
Lum	4.34	4.99	0.65	Vcan	−1.51	−2.51	−1.00
Bgn	3.21	4.14	0.94	Nid2	−0.70	−2.10	−1.40
Matrix remodeling proteins							
Serpinb3a	3.11	6.08	2.96	Adamts19	−2.48	−2.97	−0.50
Adam33	4.99	6.06	1.07	Adamts15	−1.63	−2.91	−1.27
Ctsk	4.84	5.72	0.88	Elane*	2.72	0.00	−2.72
Ctsh	4.52	5.71	1.19	Serpine2	−1.29	−2.24	−0.95
Serpinb5	1.03	4.89	3.86	Adamts9	−2.20	−0.69	1.52
Capn1	1.90	3.26	1.37	Adam11	−1.21	−1.94	−0.73
Adamts18*	3.03	1.51	−1.51	Adam19	1.08	−0.65	−1.73
Elane*	2.72	0.00	−2.72	Adamts1	−1.31	−1.58	−0.28
Adamts12	2.45	2.66	0.21	Adamts6	−0.04	−1.53	−1.49
Adamts2	1.18	2.52	1.34	Adamts18*	3.03	1.51	−1.51
Cell-ECM receptors							
Itgb4	5.94	7.36	1.42	Itga9	−1.58	−3.45	−1.87
Cd44	1.25	3.35	2.10	Itga8	−2.25	−3.30	−1.05
Ddr1	2.31	2.89	0.59	Itga4	−1.26	−1.69	−0.43
Itga11	2.53	2.72	0.19
Sdc2	2.68	2.00	−0.68
Junction proteins							
Dsp	4.94	6.33	1.39	Cdh5	−1.23	−4.74	−3.51
Dsc2	4.64	5.98	1.34	Cldn11	−2.53	−4.34	−1.80
Cldn1	3.48	5.11	1.63	Cldn5	−1.84	−4.32	−2.48
Dsc3	4.97	4.27	−0.70	Cldn6	−0.65	−2.52	−1.86
Dsg1a	2.60	4.90	2.30	Cdh2	−0.94	−2.44	−1.50
Esrp2	3.54	4.66	1.12	Gjc1	−1.03	−1.69	−0.66
Gjb2	4.19	4.64	0.45	Cdh24	−0.29	−1.26	−0.97
Emp1	3.65	4.53	0.88	Jam3	−0.67	−1.17	−0.50
Dsg2	3.55	4.06	0.50				
Cldn3	2.38	3.79	1.41				

Collagens are represented in a separate table (see Supplementary Table S8).

* These genes are upregulated and then downregulated.

**Table 3:** Top Differentially Expressed Epithelial-Associated Genes

**Symbol**	**Log2**		
	14.5/10.5*	16.5/10.5*	**16.5/14.5**
Keratins			
Krt12	6.50	10.92	4.42
Krt6a	5.15	10.33	5.18
Krt13	3.17	10.00	6.83
Krt15	6.58	9.71	3.13
Krt5	7.36	9.34	1.99
Krt14	5.45	7.99	2.54
Krt19	5.10	6.65	1.55
Krt1	3.39	4.14	0.75
Krt7	2.93	3.67	0.74
Krt8	1.24	2.08	0.84
Other epithelial genes			
Klf5	4.22	6.15	1.94
S100a6	4.26	6.06	1.79
Pax6	3.85	4.77	0.92
Apoj	3.60	4.75	1.15
Emp1	3.65	4.53	0.88
Cdh1	2.57	3.13	0.57
Glut1	0.76	1.75	0.99
Cdh3	1.69	1.33	−0.36
Tfap2a	1.60	1.58	−0.02
Gja1	1.58	1.32	−0.26

* Surface ectoderm is not included in E10.5 samples.

**Table 4:** Top Differentially Expressed Cell Cycle-Associated Genes

**Symbol**	**Log2**		
	**14.5/10.5**	**16.5/10.5**	**16.5/14.5**
Ccnd1	−3.41	−4.29	−0.88
Lin28b	−3.68	−3.68	0.00
Lin28a	−3.41	−3.41	0.00
Cdc6	−1.29	−3.32	−2.04
Mcm10	−1.41	−3.31	−1.90
Mcm5	−1.32	−3.16	−1.83
Ccne1	−1.91	−2.94	−1.03
**P21**	**−0.35**	**2.50**	**2.84**
Cdc45	−1.09	−2.83	−1.74
Mcm7	−1.41	−2.70	−1.29
Gins1	−0.85	−2.57	−1.73
Mcm3	−1.17	−2.54	−1.36
Dbf4	−0.97	−2.40	−1.43
E2f2	−1.06	−2.37	−1.31
Mcm2	−0.87	−2.33	−1.46
Mcm4	−0.90	−2.23	−1.33
Cdk1	−0.78	−2.14	−1.35
Cdc25c	−0.51	−2.07	−1.56
Mcm6	−0.87	−2.06	−1.19
Skp2	−0.97	−2.01	−1.04

Upregulated gene is highlighted in bold.

**Table 5:** Top DEGs Associated With Angiogenesis and Axon Guidance

**Symbol**	**14.5/10.5**	**16.5/10.5**	**16.5/14.5**	**A**	**AG**	**AA**
Upregulated (Log2)						
Thbs4	2.38	6.06	3.68			✓
Thbs2	4.09	5.95	1.86			✓
Pax6	3.85	4.77	0.92		✓	✓
Wnt4	4.13	4.55	0.42		✓	
Ntn4	3.74	4.46	0.71	✓	✓	
Sema3C	2.92	3.84	0.91		✓	✓
Sema5A	3.38	3.62	0.24	✓	✓	
Egfr	3.46	3.03	−0.43	✓	✓	
Plxdc2	2.16	3.13	0.97			✓
Ngf	2.84	3.09	0.24	✓	✓	
Ntf5	2.74	2.50	−0.24	✓	✓	
Epha1	2.45	2.70	0.25	✓	✓	
Ntn1	2.11	2.45	0.35	✓	✓	
Hif3a	2.33	−3.02	−5.35	✓	✓	
Sema3F	1.70	2.23	0.53		✓	✓
Vcam1	1.52	2.19	0.67	✓		
L1cam	−0.74	1.34	2.08	✓	✓	
Erbb3	0.96	2.01	1.06	✓	✓	
Efna5	1.76	1.93	0.17	✓	✓	
Wnt5a	0.47	1.85	1.38	✓	✓	✓
Downregulated (Log2)						
Rnh1	0.66	−6.02	−6.68			✓
Hif1a	−0.24	−5.60	−5.37	✓	✓	
Hyou1	−0.71	−5.49	−4.77	✓		
Hif3a	2.33	−3.02	−5.35	✓	✓	
Cadh5	−1.23	−4.74	−3.51	✓		
Tie1	−1.10	−4.21	−3.11	✓	✓	
Ebf1	−3.74	−3.74	0.00		✓	
Gata3	−3.55	−3.55	0.00	✓		✓
Hig2	0.27	−3.27	−3.54	✓		
Cxcl12	−0.80	−3.52	−2.72	✓	✓	
Gata2	−3.11	−3.26	−0.15	✓		
Tie2	−2.56	−3.19	−0.64	✓	✓	
Robo4	−1.18	−3.00	−1.82		✓	✓
Efnb3	−0.99	−2.18	−1.19	✓	✓	
Ang	0.42	−1.62	−2.03	✓	✓	
Efna2	−1.12	−1.92	−0.81	✓	✓	
Vegfc	−1.76	−1.78	−0.02	✓		
Plxa4	1.52	−0.26	−1.78	✓	✓	
Tgfβ2	0.74	−0.81	−1.55	✓	✓	
Plxd1	−0.65	−1.48	−0.83	✓	✓	✓

Checkmarks indicate potential association with pathways based on published data. A, angiogenesis; AG, axonguidance; AA, antiangiogenic.

Our data also show a high number of cell cycle genes are downregulated and cell cycle inhibitors are upregulated (Table 4, Supplementary Table S13), suggesting an overall reduction in cell proliferation. We also observed that genes involved in angiogenesis and axon guidance were differentially regulated (Table 5, Supplementary Table S14) and have potential roles in establishing the neurovascular patterns that lead to high innervation and corneal avascularity.

**Figure 7:**
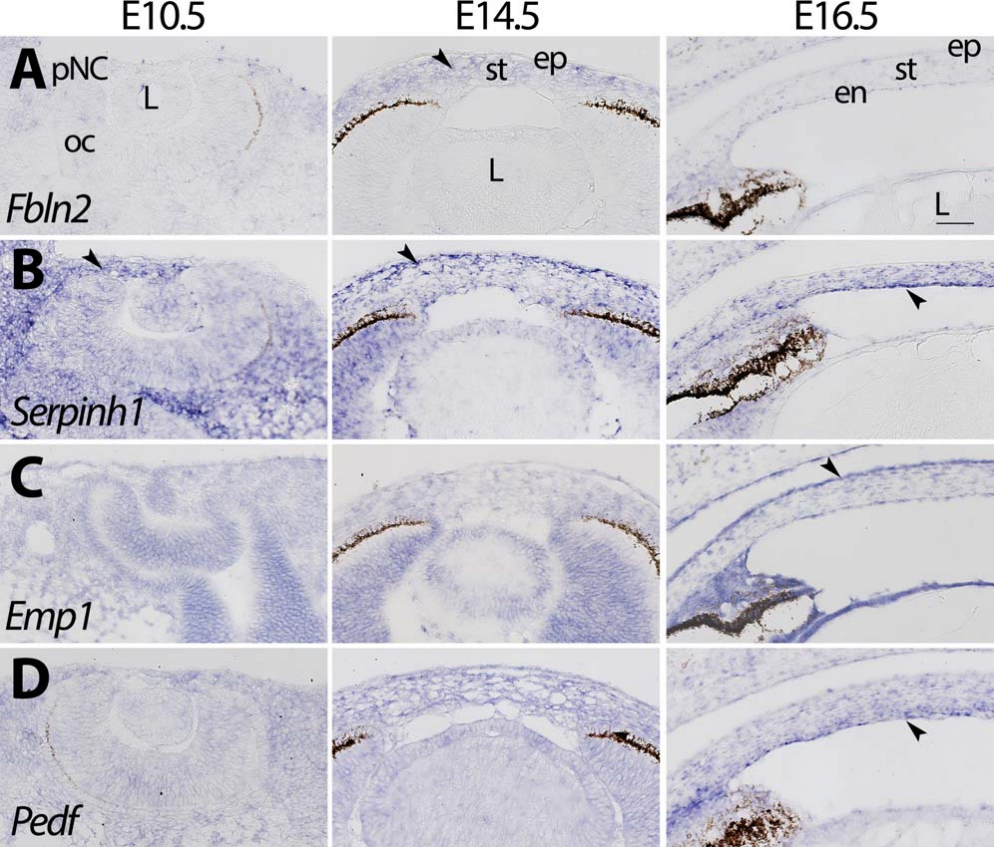
Spatiotemporal expression of genes involved in corneal morphogenesis. (A) Fbln2 is expressed at low levels at E10.5 and E16.5 and is enriched throughout the stroma at E16.5. (B) Serpinh1 is expressed in the pNC, stroma, and epithelium. At E10.5 it is enriched in the temporal mesenchyme, at E14.5 enriched in the anterior stroma, and E16.5 enriched in the endothelium. (C) Emp1 is expressed in all stages and layers, and enriched in the epithelium at E16.5. (D) Pedf is expressed in all stages and layers and enriched in the posterior cornea at E16.5. Black arrows represent regions of enriched expression. Scale bar: 50 μm.

We validated the spatiotemporal expression of several genes identified in our data. *Fbln2*, which encodes an ECM glycoprotein,^68^ is expressed at low levels in the pNC at E10.5, strongly expressed in the corneal mesenchyme at E14.5, and sparsely expressed in the stroma and endothelium at E16.5 (Fig. 7A). *Serpinh1*, which is involved in collagen biosynthesis,^69^ is expressed in the pNC at E10.5 and maintained in the corneal mesenchyme at E14.5 and in the stroma and endothelium at E16.5 (Fig. 7B). Cell junction protein *Emp1*^70^ shows broad expression at all time points but is enriched in the epithelium at E16.5 (Fig. 7C). Antiangiogenic protein *Pedf*^71^ is also broadly expressed at all time points but shows strong localization to the posterior stroma and endothelium at E16.5 (Fig. 7D).

## Discussion

Corneal development occurs during a critical period when the adjacent presumptive lens and retinal tissues undergo morphogenic changes and gene expression.^5^,^72^ These changes in the ocular environment play a crucial role in directing differentiation of both the NCC- and ectoderm-derived corneal progenitors.^34^,^72^ In this study, we provide the first detailed analysis of the transcriptome profiles of corneal cells during development. We have identified genes that are enriched at E10.5, E14.5, and E16.5, which may respectively be involved in pNC migration and proliferation, differentiation of the corneal layers, and organization of the ECM and cell-cell junctions. We link these data to genes involved in key signaling pathways and transcriptional regulation of cell behavior.

NCC contribution to the corneal endothelium and stromal keratocytes comprises the largest proportion of the cornea.^5^ Due to their dynamic and multipotential characteristics, NCCs are primed to respond to new signals from surrounding environments during their migration from the neural tube and aggregation into the periocular region.^73^ A majority of the candidate NCC genes are expressed in the periocular mesenchyme at E10.5, which could be important for maintenance of multipotency, which is required for subsequent differentiation into various ocular tissues, including the cornea, iris, and the orbital bones and cartilage.^74^,^75^ Consistent with this observation, our data revealed progressive downregulation of the NCC genes, such as *Sox9* and *Sox10*, which are involved in chondrogenesis and neural differentiation, respectively.^76^,^77^ The NCC genes that were expressed in the cornea, such as *Snai2* and *Twist1*, may either maintain their roles or take on different functions during differentiation. Twist1 is involved in craniofacial development and is an inhibitor of Sox9 and Sox10,^78^–^80^ suggesting a potential role in inhibiting these genes in the cornea.^81^ *Snai2* is sustained in the adult corneal epithelium during wound healing by TGFβ and plays a role in epithelial-mesenchymal transition,^82^ cell proliferation, migration, and differentiation,^83^ but its function in the corneal endothelium and stroma remain unclear.

RA signaling is a major factor during organogenesis of various tissues, including the central nervous system, ear, gut, heart, and the eye.^84^ RA signals in the periocular mesenchyme and presumptive cornea are either autocrine or derived from the ectoderm, optic cup, or lens.^72^,^85^ Our data indicate that both pNC and embryonic corneas have the potential for retinol uptake and RA metabolism, but these processes are strictly regulated. We observed that *Raldh3*, which is expressed in the corneal epithelium,^34^ may be the major source of RA synthesis at E16.5. All cellular RA binding proteins were significantly downregulated at E14.5. This, coupled with elevated expression of *Cyp26a1*, suggests a decrease in RA-mediated signaling. Differential expression of modulators of RA signaling is crucial for proper development of various tissues and organs.^35^ *Cyp26a1* mutant mice exhibit patterning defects in limbs and the central nervous system due to an elevation of RA signaling.^86^ Strong expression of *Cyp26a1* in the corneal epithelium suggests its involvement in moderating the RA signaling to levels that permit cell differentiation. Upregulation of RA metabolizing enzymes *Adh1* and *Adh7*, along with *Crabp1* and *Fabp5*, may represent increased signaling through alternative pathways. Crabp1-RA interaction activates Erk1/2, which triggers a signaling cascade that regulates cell cycle and promotes differentiation.^87^,^88^

TGFβ signaling has been implicated in driving cell migration and differentiation and formation of the collagen ultrastructure during corneal development.^17^,^89^ Our data show elevated *TGFβ2* transcripts concomitant with the formation of the corneal endothelium,^5^ followed by its rapid downregulation. Combined with the previous observation that the corneal endothelium is absent in TGFβ2 knockout mice,^18^ our data suggest that high levels of TGFβ2 are required for its formation. We also observed upregulation of *TGFβ3*, which stimulates matrix assembly in vitro.^90^,^91^ Upregulation of *TGFβR2* is in line with its function as the primary facilitator of TGFβ signaling. *TGFβR2* mutants recapitulate *TGFβ2* knockout mice phenotypes.^89^ In addition, they are unable to phosphorylate Smad2, misexpress Foxc1 and Pitx2, and display abnormal keratocyte differentiation and collagen synthesis.^89^ Canonically, TGFβ interacts with TGFβR2 to recruit and phosphorylate TGFβR1, which activates Smad2/3 signaling.^42^,^92^ Although the downregulation of the interacting partner TGFβR1 was unexpected, TGFβR2 can also form a complex with TGFβR3, which has higher specificity for TGFβ2.^93^,^94^ Combined with the downregulation of Smad2/3 inhibitors, this indicates an increased activity of TGFβ signaling. Along with the induction of *lumican* and *keratocan*,^18^ TGFβ signaling may mark the transition from highly proliferative pNC toward induced differentiation. Our data also show upregulation of *Aebp1* and *Creb3l1*, which are important for collagen synthesis.^47^,^48^ These genes may cooperate with other sources of collagen synthesis and maturation, such as *Bmp3* and *Bmp1*.^95^,^96^

The Wnt/β-catenin pathway is required for the proper development of the cornea.^19^,^97^,^98^ During mouse corneal development, Wnt ligands are expressed throughout the presumptive epithelium.^56^ Increased expression of *Wntless* and *Porc* indicate that Wnt signaling may also exert paracrine effects to the stroma. This is supported by reports of expression of Fzd receptors and activation of Wnt signaling in the stromal mesenchyme and corneal endothelium.^56^,^99^ Although Wnt ligands were uniformly upregulated, there was a clear distinction in the differential expression of *Fzd. Fzd4* and *Fzd10* are associated with the Wnt/β-catenin pathway, whereas *Fzd3* and *Fzd6* are involved in the Wnt/PCP pathway.^100^–^103^ *Fzd4* is required for retinal angiogenesis and implicated in corneal neovascularization.^104^,^105^ *Fzd3* is involved in neural crest induction and migration.^106^–^108^ Reduced expression of *Fzd4* and *Fzd3* and upregulation of *Fzd10* and *Fzd6* may be required for corneal cell differentiation and avascularity.^100^–^103^,^109^–^111^ Despite upregulation of Wnt ligands and receptors, our data suggest that Wnt/β-catenin signaling is inhibited at multiple levels. This complements previous observations that active Wnt/β-catenin signaling is absent in the corneal epithelium at E14.5 and E16.5, and it is progressively reduced in the stroma until postnatal day 3.^99^ This downregulation is critical for proper development of the cornea.^19^,^97^,^98^ In contrast, our data suggest that noncanonical Wnt pathways are upregulated. The Wnt/PCP and Wnt/Ca pathways have been studied during the formation of the eye field and retinogenesis,^112^ but their roles in the cornea are not clear. Our data indicate an increase in the components of the Wnt/PCP pathway, including *Wnt4*, *Wnt5a*, and *Fzd6*.^100^,^113^,^114^ In adults, the Wnt/PCP pathway is important for corneal homeostasis and also guides directional migration of epithelial cells during wound healing.^110^ Wnt/PCP signaling is also involved in cell differentiation, collagen orientation, cell alignment, and axon guidance,^115^–^117^ all of which are required for proper corneal development.

Our data suggest multiple novel connections between the RA, TGFβ, and Wnt signaling pathways. It is well established that the RA induction of *Pitx2* suppresses Wnt signaling through upregulation of *Dkk2*,^97^,^118^ and we also observe this pattern. In addition, misregulation of either Wnt or TGFβ greatly impacts Pitx2 levels, suggesting that the different pathways interact for proper signaling control.^89^,^97^ Potential crosstalk is observed in the upregulation of genes associated with RA signaling (*Sox6* and *Hic1*), which suppress Wnt signaling.^119^–^123^ The Wnt activating genes (*Prrx2* and *Hmga2*) are upregulated by TGFβ,^51^,^52^ and we observed that *Hmga2* localizes to the corneal epithelium where Wnt expression is dominant.^56^ *Strap* activates Wnt but represses TGFβ,^124^,^125^ and its downregulation may play an important role in balancing these pathways. Our data indicate that the reduction in proliferation occurs at E14.5 and progresses during corneal development. It is likely the RA and TGFβ pathways modulate the cell proliferation promoted by Wnt signaling, which may occur through regulation of *Lin28*, *C-myc*, *Id2*, and *Id3*.^45^,^97^,^126^–^129^ Proper regulation of Wnt signaling is crucial, as gain of function in epithelial β-catenin and DKK mutants show increased proliferation, impaired differentiation, and reduced ECM in the epithelium and stroma.^97^,^98^,^130^,^131^ This arrangement may change in the postnatal cornea as the epithelium undergoes stratification.^7^ Verification of these associations during corneal development will require additional studies.

The expression of ECM proteins is abundant and critical for the coordinated fibrillogenesis of the cornea. The absence of either collagens or regulatory proteoglycans causes dysfunctional fibrillogenesis and corneal opacity.^10^,^132^ Our results confirm a high expression of transcripts and upregulation of well-known corneal ECM proteins, including decorin, lumican, keratocan, and collagen I. Interestingly, several of the downregulated genes (*Vtn*, *Vcan*, *Has2*, and *Tgfbi*) are involved in neural crest induction and migration.^133^–^137^ Several matricellular genes are upregulated at E14.5 and downregulated at E16.5 (*Fbln2*, *Spp1*, and *Ecm1*), suggesting that they are required for cell migration, differentiation, or the initial organization of the corneal ECM. Upregulation of matrix remodeling genes from the cathepsin, matrix metalloproteinase, and a disintegrin and metalloproteinase families may be required for cellular positioning and collagen alignment, which are crucial for establishing a lattice structure and transparency.^138^–^140^ Genes that regulate cell junctions follow a similar trajectory and may be important for intercellular communications and establishing the epithelial and endothelial barrier.^141^ This coincides with genes that regulate epithelial differentiation and elevation of epithelial markers, suggesting that the maturation and function of the epithelium develop simultaneously.^142^,^143^ Interestingly, our data indicate that epithelial genes, such as *Emp1*, *Gsto1*, *Gsta4*, and *Glut1*, are also expressed in the pNC. This could indicate the epithelial origin of the pNC or a functional role in the mesenchyme. Some epithelial genes, such as *Slurp1* and *Psca*, are not expressed at E14.5 and E16.5, indicating that they are required at later stages of corneal development and are involved in maintenance of the epithelial layer.^144^–^146^

Cross talk between the ECM and resident cells is mediated through cellular receptors, mostly composed of integrins. Itga3b1 and Itga11b1 are required for collagen deposition and matrix assembly.^147^,^148^ ItgaVb1 and ItgaVb5 may affect neural guidance^149^ or interact with latent TGFβ, which may affect ECM assembly.^150^,^151^ Itga4b1, Itga8b1, and Itga9b1 are receptors for fibronectin, and they mediate cell adhesion and migration.^152^–^155^ Their downregulation implies reduced motility. Upregulation of *Ddr1*, which is regulated by collagen, may provide a feedback mechanism to retain high activity of matrix remodeling genes.^156^,^157^ Our data show that both *Agrin* and its receptor *Dag1* are upregulated in the cornea.^158^ Misregulation of either Agrin or Dag1 causes similar corneal defects, suggesting they may interact during corneal development.^158^–^161^

Angiogenesis and neurogenesis are two closely related processes that require intricate orchestration of signals to generate a highly innervated yet avascular cornea. Our previous studies indicated that these two processes are separated during early corneal development.^162^,^163^ Our data reveal that multiple factors common to neurovascular patterning were highly expressed or upregulated concomitantly with antiangiogenic factors. This includes the class 3 semaphorins (Sema3A, Sema3C, and Sema3F) that we studied.^162^–^166^ We also observed upregulation of an extremely potent antiangiogenic factor, *Pedf*, and its receptor *Plxdc2*.^167^ Pedf protects against neovascularization in disease and wound healing in the retina and cornea,^71^,^168^–^170^ and it is likely to play a similar role during corneal development.

## Conclusions

Here, we report the first transcriptome analysis of the early development of the mouse cornea. Our data identify a large number of differentially regulated genes during corneal development. We describe the genetic landscape of corneal morphogenesis and provide novel insights of how cross talk between the RA, Wnt, and TGFβ pathways regulates transcription factors involved in cell migration, proliferation, and differentiation. This data will serve as a valuable resource for identifying novel genes essential for corneal development and potential targets for corneal therapies.

## Supplementary Material

Supplement 1

Supplement 2
