# Supplementary material for: Transformation of the Transcriptomic Profile of Mouse Periocular Mesenchyme During Formation of the Embryonic Cornea
Source: Invest Ophthalmol Vis Sci. 2019 Feb;60(2):661–76. doi: 10.1167/iovs.18-26018 (PMC6383728; doi:10.1167/iovs.18-26018)
Supplement: Supplement 1 [file iovs-59-15-45_s01.pdf]

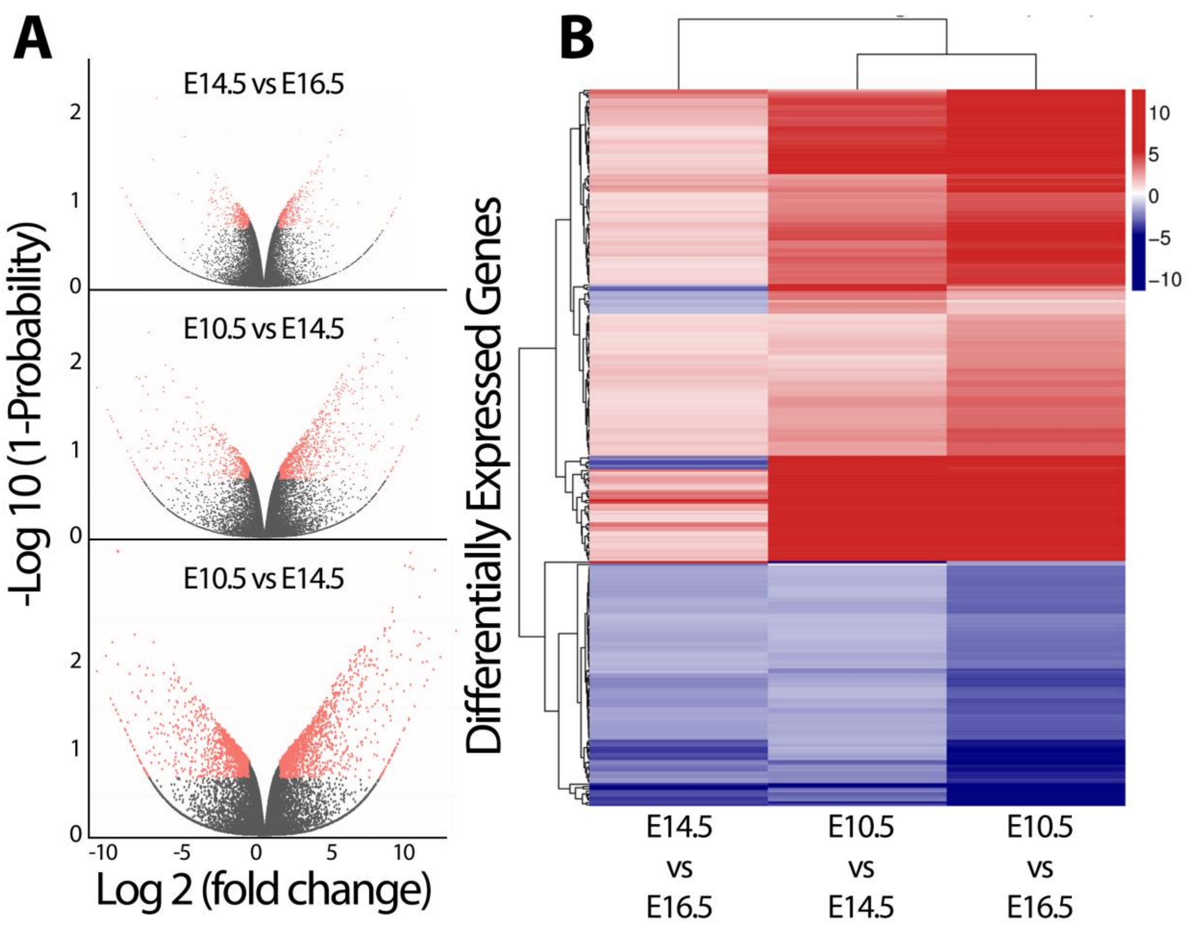

Supplementary Figure S1. Identification of DEGs and hierarchical analysis. (A) DEGs in red, are calculated based on a probability ≥ 0.8 and |(log2 fold change)| ≥ 1. (B) Hierarchical clustering analysis identifies similar gene expression patterns between the three developmental stages. Color code is based on z-score indicating red (upregulated) and blue (downregulated).
